# Ascites-Derived Organoids to Depict Platinum Resistance in Gynaecological Serous Carcinomas

**DOI:** 10.3390/ijms241713208

**Published:** 2023-08-25

**Authors:** Andrea Estrella Arias-Diaz, Miriam Ferreiro-Pantin, Jorge Barbazan, Edurne Perez-Beliz, Juan Ruiz-Bañobre, Carlos Casas-Arozamena, Laura Muinelo-Romay, Rafael Lopez-Lopez, Ana Vilar, Teresa Curiel, Miguel Abal

**Affiliations:** 1 Translational Medical Oncology Group (Oncomet), Health Research Institute of Santiago de Compostela (IDIS), University Hospital of Santiago de Compostela (SERGAS), Trav. Choupana s/n, 15706 Santiago de Compostela, Spain; andrea.estrella.arias.diaz@sergas.es (A.E.A.-D.); miriam.ferreiro.pantin@sergas.es (M.F.-P.); jorge.barbazan.garcia@sergas.es (J.B.); juan.ruiz.banobre@sergas.es (J.R.-B.); carlos.casas.arozamena@sergas.es (C.C.-A.); laura.muinelo.romay@sergas.es (L.M.-R.); rafael.lopez.lopez@sergas.es (R.L.-L.); teresa.curiel.garcia@sergas.es (T.C.); 2 Department of Medicine, Universidade de Santiago de Compostela (USC), 15782 Santiago de Compostela, Spain; 3 Centro de Investigacion Biomedica en Red de Cancer (CIBERONC), Monforte de Lemos 3-5, 28029 Madrid, Spain; 4 Department of Pathology, University Hospital of Santiago de Compostela (SERGAS), Trav. Choupana s/n, 15706 Santiago de Compostela, Spain; edurne.perez.beliz@sergas.es; 5 Department of Gynecology, University Hospital of Santiago de Compostela (SERGAS), Trav. Choupana s/n, 15706 Santiago de Compostela, Spain; ana.vilar.lagares@sergas.es

**Keywords:** gynaecological serous carcinomas, ascites-derived organoids, platinum resistance, differential gene expression analysis

## Abstract

Gynaecological serous carcinomas (GSCs) constitute a distinctive entity among female tumours characterised by a very poor prognosis. In addition to late-stage diagnosis and a high rate of recurrent disease associated with massive peritoneal carcinomatosis, the systematic acquisition of resistance to first-line chemotherapy based on platinum determines the unfavourable outcome of GSC patients. To explore the molecular mechanisms associated with platinum resistance, we generated patient-derived organoids (PDOs) from liquid biopsies of GSC patients. PDOs are emerging as a relevant preclinical model system to assist in clinical decision making, mainly from tumoural tissue and particularly for personalised therapeutic options. To approach platinum resistance in a GSC context, proficient PDOs were generated from the ascitic fluid of ovarian, primary peritoneal and uterine serous carcinoma patients in platinum-sensitive and platinum-resistant clinical settings from the uterine aspirate of a uterine serous carcinoma patient, and we also induced platinum resistance in vitro in a representative platinum-sensitive PDO. Histological and immunofluorescent characterisation of these ascites-derived organoids showed resemblance to the corresponding original tumours, and assessment of platinum sensitivity in these preclinical models replicated the clinical setting of the corresponding GSC patients. Differential gene expression profiling of a panel of 770 genes representing major canonical cancer pathways, comparing platinum-sensitive and platinum-resistant PDOs, revealed cellular response to DNA damage stimulus as the principal biological process associated with the acquisition of resistance to the first-line therapy for GSC. Additionally, candidate genes involved in regulation of cell adhesion, cell cycles, and transcription emerged from this proof-of-concept study. In conclusion, we describe the generation of PDOs from liquid biopsies in the context of gynaecological serous carcinomas to explore the molecular determinants of platinum resistance.

## 1. Introduction

High-grade serous ovarian carcinomas (HGSOCs), the most prevalent serous gynaecological tumour, along with primary peritoneal serous carcinomas (PPSCs) and fallopian tube serous carcinomas (FTSCs) constitute the most lethal gynaecological tumours and are considered a distinctive entity due to substantial histological, molecular, and clinical similarities. These malignancies are usually diagnosed at advanced stages and show limited response to current treatments, resulting in a high risk of recurrence and low 5-year survival rates. Also, with substantial similarities, the relatively rare endometrial cancer subtype of uterine serous carcinoma (USC) presents very poor prognosis when diagnosed at advanced stages.

One of the main factors contributing to the high mortality in patients with gynaecological serous carcinomas (GSCs) is the inability to detect the disease at an early stage with localised disease. Unfortunately, in 75–80% of cases, the disease has already reached an advanced stage by the time a patient becomes symptomatic, often presenting with peritoneal dissemination that significantly compromises the oncological outcome. The 5-year survival for patients diagnosed with early-stage HGSOC can reach 75–80% depending on the series, compared to only 10–30% for those presenting with an advanced-stage disease [1].

Likewise, tumour heterogeneity and rapid acquisition of resistance to conventional chemotherapeutic approaches strongly contribute to the poor outcome of patients. Gynaecological serous carcinomas are typically treated with debulking surgery followed by platinum-based chemotherapy [2]. After the diagnostic laparoscopy, GSC patients may undergo three to four cycles of chemotherapy before interval debulking surgery or they may be directly treated with primary debulking surgery, depending on the probability of achieving a complete R = 0 resection of the disease. This factor is the main clinical determinant associated with progression-free and overall survival in GSC. After surgery, chemotherapy is completed (total of 6–8 cycles), and in advanced USC cases, additional radiotherapy may be considered as an option.

Despite this radical initial treatment, the majority of GSC patients (85%) will recur with a median disease-free survival (DFS) of 18 months. Although this cut-off is debatable and clinically flexible, traditionally, patients are considered platinum-sensitive and therefore eligible for retreatment with platinum-based chemotherapy if DFS exceeds 6 months. Conversely, if DFS is shorter, patients are classified as platinum-resistant and ineligible for retreatment with platinum-based chemotherapy. Nevertheless, platinum-sensitive patients will mostly relapse within the next 2–3 years, progressing into a platinum-resistant setting that will be treated with sequential lines of chemotherapies with limited efficacy [3]. 

Regarding the molecular mechanisms associated with resistance to platinum-containing drugs, they can be categorised into several broad biological processes, including (i) regulation of drug entry, exit, accumulation, sequestration, and detoxification; (ii) enhanced repair and tolerance of platinum-induced DNA damage; (iii) alterations in cell survival pathways; (iv) alterations in pleiotropic processes and pathways, and (v) changes in the tumour microenvironment [4].

Unfortunately, in this context, disease progression often manifests as massive peritoneal carcinomatosis, which occurs due to tumour cell spread through direct extension to adjacent organs within the peritoneal cavity or through the detachment of cells from the primary tumour [5]. Recurrent disease and chemoresistance are consistently associated with the formation of ascites and multiple peritoneal implants involving the surface of the affected organs, typically colonising the mesothelial cell layer. Metastatic cells frequently appear as spheroids or aggregates of suspended tumour cells that are commonly isolated from the malignant ascites of patients with advanced disease. These aggregates are often accompanied by variable proportions of benign mesothelial cells, fibroblasts, macrophages, other immune effector cells, as well as a plethora of chemokines, cytokines, and soluble factors acting as a pro-inflammatory reservoir [6]. These cellular aggregates have been proposed as fundamental units of metastatic spread with the ability to survive in anchorage-independent conditions, thus forming a chemo-resistant niche that enables GSC cells to survive platinum-based therapies.

**Figure 1:**
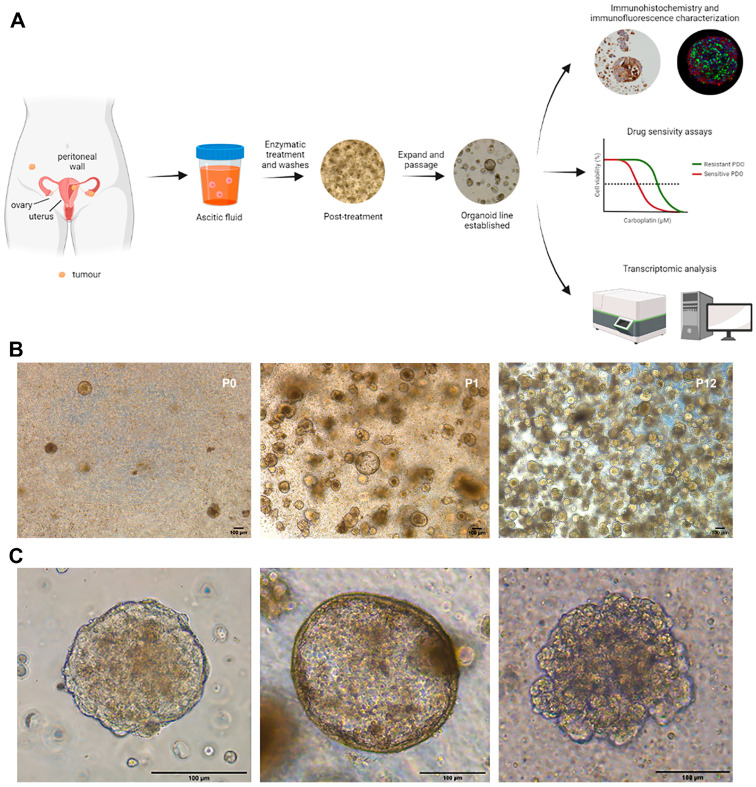
Establishment of patient-derived organoids from the ascites of GSC patients. (**A**) Scheme of the workflow and characterisation of PDOs derived from ascites. Ascitic fluid collected from GSC patients was processed and cultured as organoids, followed by immunohistochemical and immunofluorescence characterisation, evaluation of platinum sensitivity, and differential gene expression analysis. (**B**) Representative example of ASC2 organoid derivation from fresh ascites (passage 0, P0) to an established organoid line through sequential passaging (P12). Representative bright-field images at indicated passages after seeding. Scale bars, 100 μm. (**C**) Different morphologies of GSC PDOs: dense ((**left**); representative of ASC1), cystic ((**centre**); representative of ASC2), and low-cohesive ((**right**); representative of ASC3) PDOs. Representative bright-field images of individual organoids are shown. Scale bars, 100 μm.

The absence of predictive biomarkers that can inform on the most effective treatment for each patient represents a clinical priority in order to improve outcomes while minimising undesirable effects. In this regard, organoid culture technology offers a promising, expanding strategy for studying cancer and developing personalised therapeutic approaches [7]. Patient-derived organoids (PDOs) are three-dimensional dynamic tumour models that can be successfully grown from ovarian tumour tissue, ascites, or pleural fluid obtained from patients. They aid in the discovery of novel therapeutics and predictive biomarkers for ovarian cancer. These models accurately recapitulate clonal heterogeneity as well as cell–cell and cell–matrix interactions. Furthermore, they have been demonstrated to match the primary tumour in terms of morphology, cytology, immunohistochemistry, and genetics [8]. In addition, to eventually identify response-predictive biomarkers, PDOs are expected to assist in clinical decision making and provide personalised therapeutic options, particularly for patients in whom standard clinical routes have been exhausted [9]. The novelty of this work resides in approaching the acquisition of resistance to platinum-based therapy through the generation of PDOs from liquid biopsy samples (ascitic fluid and uterine aspirate) of GSC patients as the model system. For this, PDOs were generated from six GSC patients both in platinum-sensitive and platinum-resistant clinical settings, and platinum-resistant PDOs were generated from platinum-sensitive PDOs. We characterised these ascites–PDOs by immunohistochemistry and immunofluorescence, assessed their sensitivity to platinum-based therapy, and compared the gene expression profiles of PDOs derived from platinum-sensitive and platinum-resistant GSC patients using nCouter Nanostring technology (Figure 1A).

## 2. Results

### 2.1. Patient-Derived Organoids Generated from the Ascites of GSC Patients Recapitulate the Histological Features of the Tumours of Origin

Ascitic fluids samples from six GSC patients (including HGSOC, PPSC, and USC) were collected through paracentesis via percutaneous drainage, which is the most regularly used procedure for short-term symptom relief. Ascites from platinum-sensitive patients were collected before the initiation of chemotherapy (ASC2) or upon (ASC5) first-line chemotherapy. This patient presented a paradoxical response, with a decrease in tumour implants under first-line platinum-based treatment but a permanent presence of ascitic fluid and the appearance of new implants (dissociated response). Likewise, ascitic fluid from platinum-resistant patients was collected during progressive disease in a clinical platinum resistance setting: patient ASC1 progressed one month after completing first-line treatment, patient ASC3 progressed four months after completion of first-line platinum therapy, and ascites from patient ASC4 were collected at progression to the fourth line of chemotherapy. The ASC6 sample originated from the uterine aspirate of a USC patient, as an alternative liquid biopsy sample specific for endometrial cancer patients, and was considered platinum-resistant upon progression four months after the completion of platinum chemotherapy (Table 1).

Ascites were processed by consecutive centrifugations with PBS and the removal of erythrocytes through incubation with Red Cell Lysis Solution. The cellular component of the ascites, including tumour cell aggregates, was then seeded in Standard Organoid Medium (SOM, Supplementary Table S1), as described in [10]. SOM conditions showed superior performance in terms of cell viability, cell number, and size of the dense functional resulting PDOs compared to other tested organoid culture media. Furthermore, PDO derivation efficiency in SOM conditions was high (86%; PDOs were generated from six out of seven GSC patients, and data from the six successful patients are included in this work) and relatively rapid, with PDOs being developed within 1–5 weeks, thus correlating with the disease burden present in the patient at the time of collection (Figure 1B). The morphology and passaging time (split ratios of 1:3–1:4) differed between PDOs (ranging from 1 to 4 weeks), with three principal phenotypes: a densely cellular structure with well-defined cell polarisation and the absence of lumen, a low-cohesive conformation with limited cell–cell adhesion, and a cystic phenotype presenting a lumen delimited by a layer of cells (Figure 1C; see also the summary information in Table 1 and Supplementary Figure S1). All PDOs were cryopreserved and biobanked after in vitro expansion.

**Figure 2:**
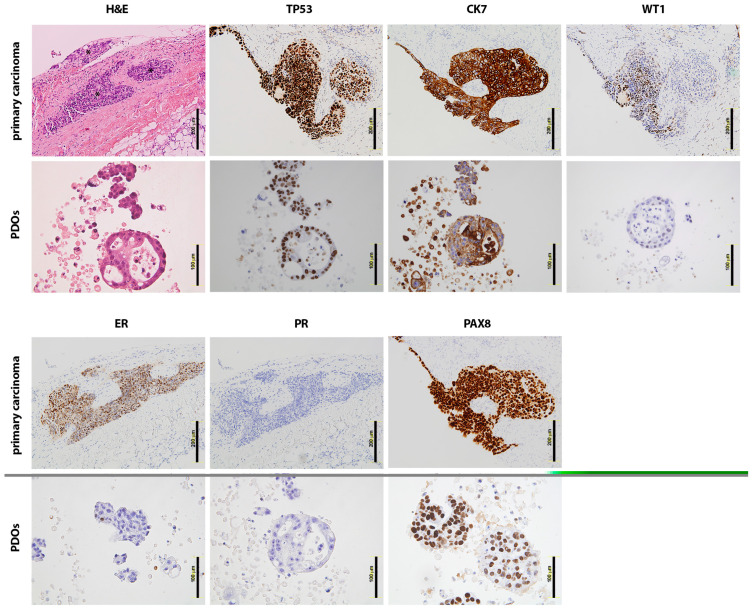
Immunohistochemistry profiling of the GSC PDOs. Histological comparison of a representative GSC PDO (ASC2) and its corresponding primary tumour tissue. The top and bottom panels show tumour tissue and organoids, respectively, which have been stained with different markers of gynaecological serous carcinomas: H&E, P53, CK7, WT-1, ER, PR, and PAX8. Abundant nuclear atypia is shown in primary tissue as well as in the PDOs. Asterisks are indicative of tumour nests in the peritoneal tissue. Scale bars: 200 μm for primary carcinomas and 100 μm for the corresponding PDOs.

Immunohistochemistry and immunofluorescence characterisation demonstrated that the established PDOs recapitulate the original tumour phenotype. Hematoxylin and Eosin (H&E) staining showed distinctive cytonuclear atypia in both the original carcinomas and the corresponding PDOs, as in the representative example from ASC2 shown in Figure 2 that is characterised by enlarged amorphous nuclei (karyomegaly, as a sign of cellular activity), a high nuclear to cytoplasmic ratio, cell and nucleoli polymorphism (angled, not rounded or with shape irregularities), hyperchromatic nuclei, and vesicular nuclei with invaginations. The expression profile of key gynaecological markers used in clinical settings, including P53, Wilms Tumour 1 (WT1), Cytokeratin 7 (CK7), PAX8, EpCAM, vimentin, and mesothelin, was also assessed by immunohistochemical (IHQ) analysis. The ASC2 PDOs exhibited positive staining for these markers, mirroring the expression patterns observed in the corresponding primary tumours and confirming the gynaecological serous carcinoma origin (Supplementary Table S2; see also Supplementary Figure S2 for representative H&E and IHQ images from ASC5 PDOs). A high frequency of P53 mutations is shared across USC (91%), HGSOC (96%), and PPSC (71.9%) [11]; consequently, the PDOs recapitulated the aberrant expression or overexpression (called “black pattern”) of P53, as shown in the representative PDOs derived from an HGSOC patient (Figure 2). CK7 and WT1 positivity are also a hallmark of PPSC, HGSOC, and USC (except for WT1 in USC, generally showing irregular positivity). As shown, the staining pattern of CK7 and WT1 in the representative PDOs is consistent and mimics the original primary carcinoma, displaying focal or patched expression in the primary carcinoma that is also represented in the corresponding PDOs (Figure 2). Likewise, the oestrogen receptor α (ERα) and progesterone receptor (PR) staining exhibited variable expression profiles depending on the patient in HGSOC and USC [12], with PPSC typically showing positive staining. As shown in the representative PDOs example, PR was found to be negative, while ERα presented a patched expression pattern similar to the corresponding primary carcinoma (Figure 2). Finally, PAX8 (Paired-Box Gene 8) showed positive expression in all the established PDOs as a marker for carcinomas of Müllerian origin [13] (Figure 2).

**Figure 3:**
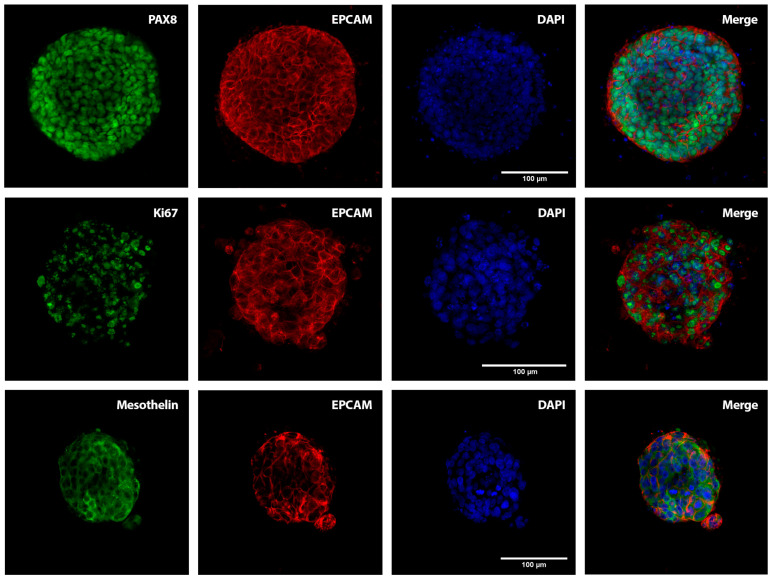
Immunofluorescence characterisation of the PDOs. Confocal microscopy images of representative PDOs derived from the ascitic fluid of GSC patients (left panels) labelled with the GSC clinical markers PAX8 (nuclear; ASC5) and mesothelin (cytoplasmic; ASC2), along with the nuclear Ki67 proliferation biomarker (ASC1). The middle panels show tumour organoids stained with the epithelial marker EpCAM and nuclear stained with DAPI; the panels on the right are merged images. Scale bar, 100 μm.

Positive nuclear PAX8 GSC marker expression was further confirmed by confocal immunofluorescence of the whole of each ascites-derived PDO, concomitant to the expression of the epithelial cell biomarker EpCAM that also illustrates the cohesive cellular structure of the ascites-derived PDOs (Figure 3; see Supplementary Figure S3 for representative examples from ASC1, ASC4, and ASC5 PDOs). We also assessed mesothelin positivity, a protein expressed in the mesothelial cell lining of the peritoneum which has been described as binding to the ovarian cancer antigen CA125 and being overexpressed in HGSOC [14], as well as the proliferative marker Ki67, which ranged from 50 to 70% in terms of positive nuclear staining (Figure 3). Residual CD45 staining, as a surrogate marker of cells from immunological and haematological origin, was indicative of the high tumoural homogeneity of the GSC PDOs (Supplementary Figure S4). Altogether, these findings indicate that patient-derived organoids generated from GSC ascites recapitulate the disease of origin both morphologically and phenotypically, mirroring the expression profile present in the primary carcinomas.

### 2.2. Sensitivity to Carboplatin in PDOs Generated from Platinum-Sensitive and Platinum-Resistant GSC Patients

**Figure 4:**
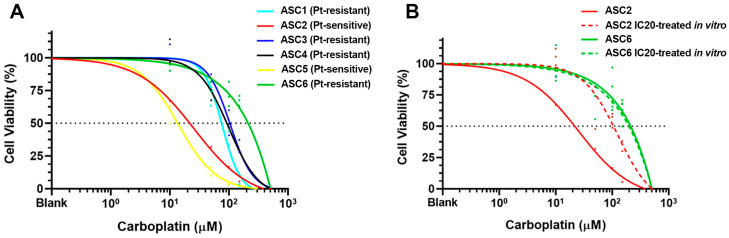
Drug sensitivity assays. (**A**) Dose–response curves of PDOs derived from two platinum-sensitive and four platinum-resistant GSC patients treated for 72 h with carboplatin. Cell viability was measured 6 days after the initiation of carboplatin treatment using the AlamarBlue assay. Results are shown as a mean of three biological replicates (except for ASC1, ASC3, and ASC4, which are shown through one biological replicate due to limited material availability), each with three technical replicates. (**B**) Platinum-sensitive PDOs derived from ASC2 (red line) were sequentially incubated with IC20 carboplatin to generate platinum-resistant PDOs (red dotted line). Notably, platinum-resistant PDOs derived from ASC6 (green line) did not result in further platinum resistance upon incubation with IC20 carboplatin (green dotted line).

To assess the sensitivity of the ascites-derived PDOs to carboplatin, we performed drug sensitivity assays with a physiological range of carboplatin. Briefly, PDOs between passages 3 and 4 from six different GSC patients were treated with concentrations of carboplatin ranging from 10 μM to 150 μM for 3 days (representative bright-field images from PDOs exposed to increased concentrations of carboplatin (10 μM to 500 μM) showing dose-dependent increased disruption and decreased viability are presented in Supplementary Figure S5). The drug was then removed and the PDOs were washed with PBS and incubated for an additional 3 days in fresh SOM before a cell viability assay (AlamarBlue) was performed. The sensitivity of each PDO to carboplatin was determined by generating dose–response curves and calculating the half-maximal inhibitory concentration (IC_50_) (Figure 4A). Of note, the PDOs originated from the ascitic fluid of GSC patients both in a platinum-sensitive and in a platinum-resistant setting, which reliably reproduced their corresponding clinical sensitivity to the standard first line of chemotherapy in vitro (Table 1). Among the six GSC patients, two were sensitive to carboplatin (ASC2 and ASC5), with IC_50_ values of 21.68 μM and 13.57 μM, respectively, while the remaining four PDOs (ASC1, ASC3, ASC4, ASC6) exhibited resistance to carboplatin (IC_50_: 75.38 μM, 103.6 μM, 92.32 μM, and 211.6 μM, respectively). On average, the PDOs derived from the ascites of GSC patients in a clinical platinum-resistant setting showed a seven-fold decrease in sensitivity to carboplatin compared to those generated from the platinum-sensitive GSC patients (Figure 4A). Although a consistent difference in carboplatin IC50 was found between the platinum-sensitive and the platinum-resistant GSC PDOs, the limited number of PDO samples resulted in a non-significant difference (*p* = 0.133; Mann–Whitney test), with a median IC_50_ of 16.35 μM for the platinum-sensitive PDOs (n = 2) while the platinum-resistant PDOs exhibited a median IC_50_ of 97.96 μM carboplatin. These results indicate that ascites-derived organoids recapitulate the sensitivity to carboplatin observed in the clinical setting.

Interestingly, we could induce carboplatin resistance in vitro through sequential exposure of the platinum-sensitive PDO ASC2 to the IC20 concentration of carboplatin. Briefly, we mimicked the standard chemotherapy regimen through sequential exposure of the PDO culture to the IC20 concentration of carboplatin for three days, followed by a recovery period in fresh SOM for one additional week before the cycle was repeated. After two cycles of chemotherapy, we evaluated the sensitivity to carboplatin and observed that the dose–response curve shifted to an increased IC_50_ of 105.3 μM, which is consistent with a platinum-resistant status (red lines in Figure 4B). Additionally, activated caspase-3 was analysed through confocal immunofluorescence to confirm the sensitivity to carboplatin treatment, specifically in the platinum-sensitive ASC2 PDOs compared to the paired platinum-resistant PDOs generated in vitro (Supplementary Figure S6). In contrast, sequential exposure of the platinum-resistant ASC6 PDO to IC20 carboplatin did not alter its sensitivity to the standard chemotherapy (green lines in Figure 4B), as was expected for an already platinum-resistant model system.

Overall, these results indicate that (i) the PDOs derived from the liquid biopsies (ascites and uterine aspirate) of six GSC patients recapitulate their immune-histochemical features and platinum sensitivity in a patient-specific manner; (ii) that sequential exposure to platinum-based therapy in the platinum-sensitive ASC2 PDOs leads to the acquisition of platinum resistance; and (iii) that GSC PDOs may represent an adequate model system to approach the molecular mechanisms underlying the acquisition of platinum resistance in the context of gynaecological serous carcinomas.

### 2.3. Comparative Transcriptomic Analysis of Platinum-Sensitive and Platinum-Resistant PDOs Generated from the Ascites of GSC Patients

To validate this hypothesis, we performed a differential gene expression (DGE) analysis comparing the molecular profile of the two platinum-sensitive PDOs (ASC2 and ASC5) and the four platinum-resistant PDOs (ASC1, ASC3, ASC4, and ASC6) using the nCouter Nanostring technology. Briefly, the PDOs were enzymatically recovered from the BME matrix, washed with PBS, and directly lysed before RNA extraction, validation of integrity and quality, and RNA quantification. Differential gene expression profiling was conducted using the nCounter PanCancer Pathways Panel, which includes 770 genes representing major canonical cancer pathways: Wnt, Hedgehog, apoptosis, cell cycle, RAS, PI3K, STAT, MAPK, Notch, TGF-β, chromatin modification, transcriptional regulation, and DNA damage control.

**Figure 5:**
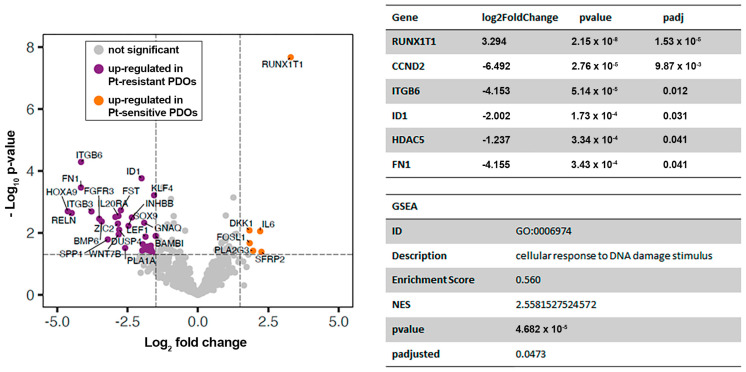
Differential gene expression (DGE) analysis between platinum-sensitive and platinum-resistant PDOs derived from the ascites of GSC patients. The volcano plot shows a Log2 fold-change and –Log10 *p*-value for the comparison of platinum-sensitive and platinum-resistant PDOs (**left panel**). The negative logFC indicates upregulation of the gene in the platinum-resistant condition. The table in the top right shows those genes presenting a significant adjusted *p*-value < 0.05. Descriptive parameters of the GSEA are also presented (**bottom right**).

After the removal of non-expressed genes using the geometric mean of negative spikes as a filter and setting of the background, data from 714 genes were normalised as described in [15]. Principal Component Analysis (PCA) showed that platinum-sensitive PDOs (n = 2) clustered together, while platinum-resistant PDOs (n = 4) showed a more scattered distribution, probably resulting from a complex clinical history with several lines of chemotherapy and possibly due to the reduced number of PDOs analysed. Genes differentially expressed between platinum-sensitive and platinum-resistant PDOs were analysed using the DESeq2 R package, resulting in 95 genes under- or overexpressed upon acquisition of platinum resistance in GSC (*p* ≤ 0.05; Supplementary Table S3), which are represented as orange and violet dots, respectively, in the volcano plot (Figure 5). Among them, RUNX1T1 (logFC = 3.294, *p* < 0.001), HDAC5 (logFC = −1.238, *p* = 0.041), ID1 (logFC = −2.002, *p* = 0.031), ITGB6 (logFC = −4.153, *p* = 0.012), FN1 (logFC = −4.155, *p* = 0.041), and CCND2 (logFC = −6.492, *p* = 0.010) showed statistically significant adjusted *p*-values and log-fold changes. The negative logFC indicates upregulation of the gene in the platinum-resistant condition. In addition, gene set enrichment analysis (GSEA) was performed for the Gene Ontology knowledgebase, obtaining statistically significant results. The biological process related with cellular response to DNA damage stimuli was found to be significantly upregulated (*p* = 0.047) in the platinum-sensitive PDOs compared to the platinum-resistant ones (NES = 2.558; Figure 5).

These findings, obtained in a limited number of samples with a cancer target gene panel, validate the promising strategy of performing comparative transcriptomic analysis in PDOs derived from the ascitic fluid of GSC patients in platinum-sensitive and platinum-resistant settings. In addition to demonstrating the reliability of this preclinical model, our analysis highlights the potential molecular mechanisms involved in the acquisition of platinum resistance, guaranteeing further research based on this organoid model system.

## 3. Discussion

The acquisition of resistance to therapy is emerging as the main challenge in the era of precision oncology to continual progression of the chronicity of cancer, and resistance to platinum, the standard therapy in GSC, is paradigmatic of this challenge. HGSOCs initially show a high response rate to DNA-damaging platinum agents such as cisplatin and carboplatin, with 85% of patients presenting sensitivity. However, after this initial response, the majority of patients eventually develop platinum-resistant disease, leading to relapse even after debulking surgery plus adjuvant therapy [16]. Similarly, after platinum/taxane-based adjuvant chemotherapy, recurrent USC is less responsive to chemotherapy compared to advanced endometrioid subtypes. 

The mechanisms leading to intrinsic/acquired platinum therapy resistance remain a significant clinical question. Novel therapeutic approaches include the use of Poly(ADP-ribose) polymerase (PARP) inhibitors, which have shown survival benefits for patients with homologous recombination deficiencies, particularly those with BRCA1/2, RAD51, RAD51D, and PALB2 mutations. The inherent extreme genomic instability and intratumoural heterogeneity in ovarian cancer [17] provide the ideal setting for adaptation and treatment escape and have been shown to drive the accelerated acquisition of multidrug resistance [18]. Evidence in HGSOC cell lines suggests that relapse is not caused by the linear acquisition of genetic alterations, but rather by treatment-induced selection and expansion of intrinsically resistant clones [19]. 

Platinum resistance is associated with various molecular mechanisms, including alterations in drug efflux, changes in intracellular proteins that bind and sequester platinum, and dysregulated expression of pro-survival or anti-survival proteins. Also, considering that the main target of platinum drugs is DNA, the sensitivity/resistance to these drugs is affected/modulated by the ability of cells to recognise and repair the DNA drug-induced damage. Specifically, there is preclinical evidence suggesting how the presence or absence of a specific DNA repair pathway (due to mutations, deletion, or epigenetic changes in genes involved in DNA repair) is associated with sensitivity/resistance to platinum drugs [20]. All this evidence points to a variety of different mechanisms supporting the resistance to platinum in a diversity of preclinical and clinical ovarian cancer models.

To advance our understanding of the histopathology and molecular features of gynaecological carcinomas, as well as the cellular origins of these cancers, several clinically relevant experimental models have been developed [21]. Among them, patient-derived organoids are emerging as a highly reliable preclinical model for studying therapeutic response [22]. Seminal work on the generation and characterisation of PDOs in ovarian cancer has already explored their use in the screening of response to the gold standard platinum-based therapy [23] or their sensitivity to PARP inhibitors based on their DNA repair profiling [24]. By contrast, integration of the tumour microenvironment in the PDO models remains a challenge and represents a current object of study in terms of improving drug screening, particularly when used in targeted therapy and immunotherapy to guide therapeutic decisions [25]. Of particular interest, PDOs derived from malignant ascites and pleural effusions, recapitulating tumour histological features, have also been used for empirical drug testing of novel therapeutics and for RNA sequencing analysis [26]. In this work, we applied this concept to investigate the acquisition of resistance to platinum-based therapy in GSC. 

Our results, comparing the transcriptomes of platinum-sensitive and platinum-resistant PDOs, point to an altered cellular response to DNA damage stimuli as the main gene ontology biological process associated with the acquisition of resistance to the first-line therapy in ovarian cancer. This aligns with extensive research on platinum resistance, with DNA damage response comprising several functional layers including sensors (e.g., MRN complex, RPA, ATRIP), signalling kinases (e.g., ATM, ATR), damage mediators (e.g., 53BP1, BRCA1/2, H2AX), downstream kinases (e.g., CHK1/2), and cell cycle checkpoint effectors (e.g., P53, P21, WEE1). Defects at each of these levels have been reported to regulate sensitivity to cisplatin in a variety of cancers, including gynaecological serous carcinomas. 

Additionally, we identified genes involved in cell adhesion (ITGB6, FN1) among those differentially expressed between platinum-sensitive and platinum-resistant PDOs, with an adjusted *p*-value < 0.05. Several studies describe the interaction of FN1 (as part of the extracellular matrix) and integrin receptors on the cell membrane, leading to cell adhesion-mediated drug resistance [27,28]. Moreover, activation of the Akt signalling pathway induced by FN1 interactions has been associated with platinum resistance in ovarian cancer cells in direct contact with cancer-associated mesothelial cells [29]. Notably, platinum-resistant cells’ ability to resolve platinum-induced DNA damage, increased dissemination in the peritoneal cavity, and adhesion at distant sites have been suggested as key properties of platinum-resistant cells [30].

The inhibitor of DNA binding 1 (ID1), significantly increased in our platinum-resistant PDOs, has been described to induce autophagy and chemoresistance through the STAT3/ATF6-mediated signalling pathway in ovarian cancer [31]. Moreover, the crosstalk between Jagged1/Notch and JAK/STAT3 signalling pathways may promote the aberrant occurrence of epithelial-to-mesenchymal transition, further reinforcing the invasion and migration abilities of platinum-resistant ovarian cancer [32]. Also related to the genes identified in the platinum resistance setting in this work, the downregulation of the transcription factor RUNX1T1, and its associated upregulation of Hypoxia-inducible factor 1α (HIF1α), has been associated with the severity and drug resistance in glioblastoma [33]. By modulating HDACs, RUNX1T1 regulates histone deacetylation, leading to transcription silencing. In this regard, the recruitment of HDAC5 has been described to modulate the novel tumour suppressor IFFO1 that inhibits tumour metastasis and reverses drug resistance in ovarian cancer [34]. The eventual correlation between RUNX1T1 and HDAC5 in the acquisition of platinum resistance in ovarian cancer should be studied in detail. Finally, the upregulation of CCND2 in platinum-resistant PDOs suggests the involvement of cyclins and cyclin-dependent kinases as principal regulators of platinum resistance in ovarian cancer [35]. This non-genetic mechanism of resistance to platinum chemotherapy involves the cell cycle stage at the time of exposure, impacting how cells respond to cisplatin [36].

In conclusion, our work sheds light on the molecular mechanisms underlying platinum resistance, and validates ascites-derived organoids as a reliable model system to progress the understanding and treatment of gynaecological serous carcinomas. As mentioned, the reduced number of GSC PDO models used in this proof-of-concept study represents its main limitation; we are currently expanding our cohort of GSC PDOs, we are increasing the matched platinum-sensitive and platinum-resistant PDO models in vitro, and we are also collecting ascitic fluid from GSC patients at the initial platinum-sensitive stage and at the platinum-resistant stage upon disease progression. All this will serve to validate our approach and expand upon the identified molecular pathways in order to develop effective strategies for overcoming platinum resistance and improving patient outcomes.

## 4. Materials and Methods

### 4.1. Establishing Organoid Cultures from Ascitic Fluids

Ascitic fluids were collected from patients through paracentesis via percutaneous drainage. The study was approved by the Galician Research Ethics Committee (reference number 2017/538) and written informed consent was obtained from all participating patients before their enrolment in the study.

Freshly obtained ascites samples were collected (20–50 mL) and refrigerated at 4 °C under sterile conditions until the procedure, with a maximum storage time of 2 h. The ascitic fluid was centrifuged at 500× *g* for 10 min at 4 °C, the supernatant was discarded, and the pellet was then resuspended in 10 mL of cold PBS for sample washing. If a visible red pellet indicative of the presence of erythrocytes was observed, the pellet was resuspended and incubated with 5 mL of Red Cell Lysis Solution (MTC096H; Biosearch Technologies, Sausalito, CA USA) for 10 min at 37 °C. After incubation, 5 mL of cold PBS was added and further centrifuged at 500× *g* for 5 min at 4 °C. The resulting pellet was further washed in 10 mL of cold PBS. Manual cell counting was performed using a Neubauer chamber. The sample was divided into two tubes: one for biobanking and the other for culturing. For cryopreservation, the pellet containing 10^6^ cells was resuspended in FBS 10% DMSO and stocked at −80 °C. For culturing, the pellet was resuspended in a mixture of 30% SOM and 70% Cultrex Basement Membrane Extract (BME; Thermo Fisher Scientific, Waltham, MA, USA). The tube was kept on ice to maintain the BME in a liquid state. A volume of 40 μL of the mixture containing 200,000–300,000 cells was plated per droplet in a prewarmed 24-well plate. After 5 min of incubation at 37 °C, the plate was inverted and incubated for an additional 25 min to allow the BME to solidify and ensure the complete distribution of the cells. Finally, 600 µL of prewarmed SOM was added to each well and incubated at 37 °C and 5% CO_2_, with SOM refreshed every 2–3 days.

Alternatively, ASC6 PDOs were established from a sample of uterine aspirate. For this purpose, a representative sample collected during surgery with a Cornier cannula was homogenised with an equal volume of cold PBS by pipetting and centrifuged at 2500× *g* for 20 min at 4 °C. The pellet was minced into small pieces using an iris scissor and transferred to a 50 mL centrifuge tube with 10 mL of cold PBS before then being homogenised with a serological pipette. Next, the sample was filtered through a 40 μm filter and the flow-through discarded; the filter was inverted and washed with 5 mL of PBS to recover the material that was stuck. After centrifugation at 500× *g* for 5 min at 4 °C, 5–8 mL of Red Cell Lysis Buffer was added and incubated for 10 min at 37 °C. To ensure lysis of the erythrocytes, the tube was flicked and 10 mL of PBS was added. The sample was homogenised with a serological pipette and centrifuged at 500× *g* for 5 min at 4 °C. The pellet was disaggregated in Digestion Medium (DMEM-F12, 1.25 U/mL Dispase II, 0.4 mg/mL Collagenase IV; Gibco), followed by incubation at 37 °C in a shaker at 1200 rpm for 40–60 min. The sample was centrifuged at 500× *g* for 5 min at 4 °C, and the pellet was resuspended in prewarmed TrypLE, followed by an incubation for 5 min at 37 °C. Next, it was passed through a 1 mL syringe 19G/21G needle 20 times in each needle at room temperature. Subsequently, 5 mL of PBS 5% FBS supplemented with 10 μM of Rock Inhibitor Y27632 was added and the sample was filtered through a 70 um cell strainer. After centrifugation at 500× *g* for 5 min at 4 °C, the pellet was resuspended in 1 mL of PBS 5% FBS/RI for manual counting using a Neubauer chamber. Finally, it was centrifuged at 500× *g* for 5 min at 4 °C and the pellet was resuspended in a mixture of 30% SOM and 70% Cultrex Basement Membrane Extract (BME), following the same protocol as described for ascitic fluid.

Serial passages were conducted by adding 500 μL of cold PBS to each well and scrapping the well with a pipette tip for 20 s. We then collected the PBS along with the disrupted domes and transferred them to a 15 mL centrifuge tube. We washed the wells with 1 mL of cold PBS to ensure that all organoids had been recovered. Next, we centrifuged at 500× *g* for 5 min at 4 °C, removed the supernatant, and added 1 mL of TrypLE (Thermo Fisher Scientific), a cell dissociation reagent. This was incubated at 37 °C for 5–10 min (depending on whether the PDOs were low-cohesive, cystic, or dense). We the pipetted up and down to break down the clusters, added cold PBS to a final volume of 10 mL, and centrifuged at 500× *g* for 5 min at 4 °C. Finally, we removed the supernatant and resuspended the pellet in the BME/SOM mixture for plating, as described above.

### 4.2. Immunohistochemical Analysis

For IHQ analysis, 1 mL of 4% paraformaldehyde (PFA) was added to each well and the plate was incubated at room temperature for 30 min. We collected the sample in a 15 mL centrifuge tube and added 5 mL of 4% PFA. This was incubated overnight at 4 °C. The following day, it was centrifugated at 500× *g* for 10 min at 4 °C. The supernatant was then discarded, and the pellet was resuspended in 1 mL of PBS and transferred to a low-binding 1.5 mL tube. It was centrifugated at 500× *g* for 10 min at 4 °C. Simultaneously, 1% agarose–PBS was heated and allowed to cool down to 60 °C. The supernatant was discarded once again and the pellet was resuspended in 1% agarose–PBS. The resuspended fixed PDOs were then transferred to cryomolds, and once the agarose had cooled down, the cassette was filled up. It was incubated for 15–30 min at room temperature to solidify and was finally stored in 70% ethanol until the staining procedure. The sections were subjected to H&E, Wilms Tumour 1, P53, PAX8, Cytokeratin 7, oestrogen receptor, and progesterone receptor staining, as described in [37]. 

### 4.3. Immunofluorescence Analysis

For this purpose, organoids were grown in BME on eight-chamber slides (ibidi GmbH, Gräfelfing, Germany) and fixed in 4% paraformaldehyde for 1 h. They were permeabilised with permeabilisation buffer (PBS, 1% triton X-100) for 1 h and blocked with blocking buffer (PBS, 1% BSA, 3% fetal bovine serum, 0.2% triton) for 1 h. They were then incubated with the primary antibodies (PAX8 1:100, EpCAM 1:500, mesothelin 1:100, Ki67 1:200, vimentin 1:200; see Supplementary Table S4) and diluted in the working buffer (PBS, 0.1% BSA, 0.3% foetal bovine serum, 0.2% triton X-100) overnight at room temperature in the dark. After several washes with the working buffer, they were incubated with the secondary antibody (goat anti-rabbit; 1:1000) for 1 h (corresponding negative controls are shown in Supplementary Figure S7). The working buffer was discarded, and the removable part of the chamber was taken off. Finally, a few droplets of Aqua-Poly/Mount (18606-100, Polysciences, Warrington, PA, USA) were added and a glass cover was placed on top. Confocal microscopy pictures were taken and images were processed with ImageJ software 2.2.0/1.54f; https://imagej.net/ij.

### 4.4. Drug Sensitivity Assay

To assess the sensitivity of the different GSC PDOs to platinum-based therapy, the PDOs were plated in 18.8 μL domes in a prewarmed 96-well plate and, when the organoids were fully formed and confluent, treated with increasing concentrations of carboplatin in technical triplicates: 10 μM, 50 μM, 100 μM, 150 μM, and 500 μM, as described in [38,39]. Three technical replicates were not treated (considering that the vehicle of the drug was water) and were interpreted as the negative control. Carboplatin was diluted in SOM without the Y27632 Rock inhibitor to avoid its improved survival and reduced apoptosis effects. The organoid cultures were treated with this concentration series of carboplatin for 3 days, and cell viability was assessed 6 days after the drug was added using the AlamarBlue assay. After 3 h of incubation with the reagent diluted in DMEM-F12 (mixture 1:10), absorbance was measured and the data were analysed with GraphPad Prism software (v. 8.4.2). Normalisation was carried out using the positive and negative controls. To analyse the data, the equation model used was “Absolute IC50, X is concentration”.

### 4.5. Nanostring nCounter Expression Assay

Differential gene expression profiling between platinum-sensitive and platinum-resistant PDOs was evaluated through the SPRINT nCounter system (NanoString Technologies, Seattle, WA USA) and the nCounter PanCancer Pathways Panel. Once the PDOs were fully formed and the culture was confluent, we discarded the media and added 500 μL of Dispase II (Thermo Fisher Scientific) (2 mg/mL PBS) to each well to allow for organoid isolation from the BME. After incubation for 15 min at 37 °C, the suspension was transferred to a 15 mL centrifuge tube. We added 100 μL of EDTA (0.5 M) for every 1 mL of dispase used and filled the tube up to 10 mL with PBS. It was then centrifuged at 300× *g* for 5 min at room temperature. The supernatant was discarded and we added 5 mL of PBS for washing. After centrifugation (500× *g* for 10 min at room temperature), the pellet was directly resuspended in Buffer RLT Plus for lysis before RNA extraction using the AllPrep DNA/RNA Mini Kit (Qiagen, Hilden, Germany). RNA quantity and quality were assessed using the TapeStation System (Agilent, Santa Clara, CA USA), with RNA Integrity Numbers (RINs) greater > 9. We used the standard gene expression protocol for RNA hybridisation with 7 μL of RNA as input, and a hybridisation time of 18 h was used for all samples. We first carried out a quality control (QC) check of the raw data and checked some technical parameters by following the manufacturer’s recommendations to ensure the absence of technical problems. All samples passed the technical QC check.

### 4.6. Differential Gene Expression Analysis

To perform differential gene expression (DGE) analysis with gene counts obtained from nCounter Nanostring, we first filtered out underexpressed genes using the geometric mean of ERCC negative spike-ins as the cut-off. We then established a data background for the filtered dataset by identifying genes that showed no significant difference between our two conditions (*p* > 0.1, baseMean > 100, |log2FoldChange| < 0.25) using the RUVg function of the RUVSeq (v1.30.0) R package [15]. DGE analysis was performed with the DESeq2 (v1.36.0) package, with the platinum-sensitive condition serving as the reference comparator. A volcano plot was generated using the EnhancedVolcano (v1.14.0) R package. Those genes with a nominal *p*-value < 0.05 in the DGE analysis were used to conduct gene set enrichment analysis (GSEA) with the clusterProfiler (v4.7.1.002) R package. All *p*-values were two-sided, and those less than 0.05 were considered statistically significant. The Benjamini–Hochberg procedure was employed to control the false discovery rate in the case of multiple comparisons. All statistical analyses were performed using R version 4.2.2 (Vienna, Austria).

## Figures and Tables

**Table 1:** Overview of GSC patients included in the study, samples, and PDOs.

Sample Code	Diagnosis ^a^	Lines of Therapy	End of Treatment	Date of Progression	Date of Collection of the Sample	Organoid Morphology
ASC1	HGSOC IV	1st line: CarboTaxol 2nd line: Niraparib	07/2022 02/2023	08/2022	25/02/2022	Dense/low-cohesive
ASC2	HGPPC	1st line: CarboTaxol 2nd line: Bevacizumab	09/2022 10/2022	-	29/03/2022	Dense/cystic
ASC3	HGSOC IIIB-IV	1st line: CarboTaxol 2nd line: Caelyx 3rd line: Paclitaxel	09/2022 01/2023 04/2023	11/2022	15/12/2022	Dense
ASC4	(OA) IIIB	1st line: CarboTaxol 2nd line: Carbo-Caelyx 3rd line: Niraparib 4th line: Carboplatin	01/2021 12/2021 05/2022 06/2022	09/2021	12/08/2022	Dense/low-cohesive
ASC5	USC IV	1st line: CarboTaxol	07/2022	Dissociated response	13/12/2022	Dense
ASC6	USC III	1st line: CarboTaxol 2nd line: CarboTaxol	02/2022 12/2021	06/2022	^b^ (05/08/2021)	Dense

^a^ HGSOC, high-grade serous ovarian cancer; HGPPC, high-grade primary peritoneal cancer; USC, uterine serous carcinoma. ^b^ In this case, the organoid line was established from a sample of uterine aspirate.

## Data Availability

Research data is available under request.

## Supplementary Materials

The following supporting information can be downloaded at: https://www.mdpi.com/1422-0067/24/17/13208/s1.
