# Supplementary material for: Ascites-Derived Organoids to Depict Platinum Resistance in Gynaecological Serous Carcinomas
Source: Int J Mol Sci. 2023 Aug 25;24(17):13208. doi: 10.3390/ijms241713208 (PMC10487816; doi:10.3390/ijms241713208)

SUPPLEMENTARY TABLES

Supplementary Table S1. Standard Organoid Medium [10] recipe (for a total of 50 mL)

Compound	Supplier	Volume Added (mL)	Stock Concentration	Final Concentration
**DMEM/F12**	Merck Millipore (D8437)	46.737	-	-
**B27**	Thermo Fisher Scientific (15360284)	1000	50X	1X
**Anti/anti**	Thermo Fisher Scientific (15240062)	500	100X	1X
**N2**	Thermo Fisher Scientific (17502001)	500	100X	1X
**Glutamax**	Thermo Fisher Scientific (35050061)	500	100X	1X
**ITS**	Thermo Fisher Scientific (41400045)	500	100X	1X
**N-acetylcysteine**	Sigma-Aldrich (A7250)	101	613 nM	1.24 nM
**Noggin**	Vitro (SIB-10267-H08H-1)	55	100 µg/mL	0.11 µg/mL
**Rspo1**	Thermo Fisher Scientific (17189851)	55	200 µg/mL	0.22 µg/mL
**SB 2021190**	Thermo Fisher Scientific (J60950MF)	16.6	20 mM	6.64 µM
**Nicotinamide**	Sigma-Aldrich (72340)	12.4	8 M	1.98 mM

**b-Estradiol**	Sigma-Aldrich (E2257)	2	25 µM	1 nM
**EGF**	PeproTech (AF-100-15)	2.5	1000 µg/mL	0.05 µg/mL
**FGF**	PeproTech (100-18B)	1	100 µg/mL	2 ng/mL
**A83**	Sigma-Aldrich (SML0788)	1	23.72 mM	474.4 nM
**Y27632**	MedChemExpress (HY-10583)	16.6	10 mM	3.32 nM

Supplementary Table S2. Overview of Immunofluorescent Profiling of Patient Derived Organoids

Sample Code	PAX8	EpCAM	Vimentin	Mesothelin
ASC1	+	+	+	+
ASC2	+	+/-	+/-	+
ASC3	N/A[a]	N/A	N/A	N/A
ASC4	+/-	-	+/-	-
ASC5	+	+	+	+
ASC6	N/A	-	+/-	N/A

+, positive expression; +/-, some positive cells; -, negative expression.

a Data non available because of insufficient material.

Supplementary Table S3. Genes differentially expressed between platinum-sensitive and platinum-resistant PDO. Positive logFC indicates up-regulation of the gene in the platinum-sensitive condition.

Gene	log2FoldChange	pvalue	padj
**RUNX1T1**	3.294	2.15E-08	1.53E-05
**CCND2**	-6.492	2.76E-05	9.87E-03
**ITGB6**	-4.153	5.14E-05	0.012
**ID1**	-2.002	1.73E-04	0.031
**HDAC5**	-1.237	3.34E-04	0.041
**FN1**	-4.155	3.43E-04	0.041
**KLF4**	-1.558	0.001	0.062
**NPM1**	1.259	0.001	0.064
**CDC14B**	-0.959	0.001	0.064
**ITGB3**	-3.783	0.002	0.121
**FST**	-2.738	0.002	0.121
**HOXA9**	-4.624	0.002	0.121
**RELN**	-4.485	0.002	0.125
**INHBB**	-2.347	0.003	0.126
**CDH1**	-0.862	0.003	0.126
**IL20RA**	-2.823	0.003	0.126
**GNGT1**	-2.933	0.003	0.126
**RFC4**	0.989	0.003	0.126
**FGFR3**	-3.506	0.004	0.132
**BMP6**	-3.418	0.004	0.153
**GNAQ**	-1.91	0.005	0.156
**PIK3R3**	-0.857	0.005	0.156
**ZIC2**	-2.844	0.005	0.156
**TET2**	-1.093	0.005	0.158
**SOX9**	-2.467	0.006	0.162
**MAP3K20**	-1.039	0.006	0.162
**DUSP4**	-2.799	0.008	0.201
**DKK1**	1.829	0.008	0.202
**MRPS5**	-0.456	0.008	0.202
**IL6**	2.209	0.009	0.203
**HES5**	1.364	0.011	0.235
**POLE2**	1.319	0.011	0.235

**WNT7B**	-2.816	0.011	0.235
**CACNA2D1**	-1.461	0.012	0.249
**BAMBI**	-1.504	0.013	0.249
**LEF1**	-1.862	0.013	0.256
**SMC1A**	0.959	0.014	0.271
**MET**	0.978	0.017	0.292
**SPP1**	-3.203	0.016	0.292
**MLLT3**	-1.406	0.017	0.292
**TMUB2**	-0.918	0.017	0.292
**ERBB2**	-1.351	0.018	0.295
**EZH2**	0.977	0.018	0.295
**ID2**	-1.362	0.02	0.313
**CHUK**	0.844	0.02	0.313
**JAK1**	0.833	0.022	0.313
**PIK3R2**	-0.894	0.022	0.313
**FOSL1**	1.836	0.021	0.313
**BCL2L1**	-0.929	0.021	0.313
**ERCC2**	-1.385	0.023	0.317
**IL1RAP**	-0.744	0.024	0.317
**IRS1**	-1.961	0.023	0.317
**COL1A1**	-1.678	0.04	0.332
**FANCC**	-0.644	0.04	0.332
**ITGA6**	-1.67	0.033	0.332
**ALKBH2**	0.883	0.037	0.332
**LEPR**	-1.324	0.039	0.332
**SPOP**	-1.152	0.031	0.332
**APH1B**	-1.173	0.028	0.332
**CDC6**	1.216	0.034	0.332
**GPC4**	-1.075	0.033	0.332
**CDC25A**	1.108	0.027	0.332
**COL2A1**	-1.909	0.035	0.332
**RAF1**	0.646	0.035	0.332
**WNT5A**	-1.633	0.039	0.332
**CDC7**	0.819	0.04	0.332
**PKMYT1**	0.913	0.039	0.332
**FANCG**	1.075	0.029	0.332

**MAP3K13**	-1.233	0.028	0.332
**CDKN2B**	-0.963	0.034	0.332
**E2F1**	1.04	0.038	0.332
**MECOM**	1.327	0.037	0.332
**MYCN**	-1.29	0.029	0.332
**SMC3**	0.664	0.036	0.332
**NOG**	-1.794	0.036	0.332
**LAMC2**	-1.676	0.026	0.332
**FOXO4**	-0.844	0.04	0.332
**PLA2G3**	1.958	0.038	0.332
**PLA1A**	-2.586	0.031	0.332
**HELLS**	1.295	0.034	0.332
**HOXA10**	-1.343	0.036	0.332
**FANCE**	1.003	0.032	0.332
**WNT10A**	-1.791	0.027	0.332
**PRDM1**	-1.977	0.037	0.332
**SFRP2**	2.256	0.041	0.335
**TGFBR2**	-0.624	0.041	0.336
**UBE2T**	0.976	0.042	0.337
**MRM2**	0.838	0.044	0.346
**DLL1**	-0.932	0.044	0.348
**LAT**	-1.617	0.047	0.349
**CHEK1**	1.262	0.047	0.349
**SETBP1**	-1.364	0.046	0.349
**NOTCH2**	-0.631	0.046	0.349
**EGFR**	-0.828	0.045	0.349
**ERCC3**	0.744	0.049	0.364

Supplementary Table S4. Antibodies (Immunofluorescence)

Reagent	Supplier
PAX8 Rabbit Polyclonal Antibody	ProteinTech (20336-1-AP)
Vimentin (D21H3) XP® Rabbit mAb (Alexa Fluor® 647 Conjugate)	Cell Signaling (9856)
EpCAM (VU1D9) Mouse mAb (Alexa Fluor® 594 Conjugate)	Cell Signaling (7319)
Mesothelin Polyclonal Antibody	Invitrogen (PA5-21662)
Ki-67 (D3B5) Rabbit mAb (Alexa Fluor® 488 Conjugate)	Cell Signaling (11882)
Anti-Hu CD45 Alexa Fluor® 647	Exbio (A6-222-T025)
Goat Anti-Rabbit IgG H&L (Alexa Fluor® 488)	Abcam (ab150077)
NucBlue™ Live ReadyProbes™ Reagent (Hoechst 33342)	Invitrogen (R37605)
Cleaved Caspase-3 (Asp175) Antibody	Cell Signaling (9661)

SUPPLEMENTARY MATERIALS AND METHODS

Immunofluorescence analysis of Cleaved Caspase-3 Antibody

Organoids were grown in BME/SOM domes (30µL) on an eight-chamber slide (Ibidi). 500µL of 10µM carboplatin, diluted in SOM without Y27632, was added to four wells, and 500µL of SOM without Y27632 was added to the other four wells (as negative controls for the drug). The slide was incubated for 24 hours. Next, the media was removed and the PDOs were fixed in 4% paraformaldehyde for 1 hour. They were permeabilized with permeabilization buffer (PBS, 1% triton X-100) for 1 hour and blocked with blocking buffer (PBS, 1% BSA, 3% fetal bovine serum, 0.2% triton) for 1 hour. Then, they were incubated with the primary antibody (Cleaved Caspase-3 Antibody) diluted in the working buffer (PBS, 0.1% BSA, 0.3% fetal bovine serum, 0.2% triton X-100) overnight at room temperature in the dark. After several washes with the working buffer, the wells incubated with Cleaved Caspase-3 previously were incubated this time with the secondary antibody (Goat Anti-Rabbit IgG H&L Alexa Fluor® 488) for 1 hour. The working buffer was discarded, and the removable part of the chamber was taken off. Finally, a few droplets of Aqua-Poly/Mount (18606-100, Polysciences) were added and a cover glass was placed on top. Confocal microscopy pictures were taken (oil, 40x), and images were processed with ImageJ software.

Immunofluorescence analysis of EpCAM and CD45

Organoids were grown in BME on eight-chamber slides (Ibidi). The media was removed and the PDOs were fixed in 4% paraformaldehyde for 1 hour. They were permeabilized with permeabilization buffer (PBS, 1% triton X-100) for 1 hour and blocked with blocking buffer (PBS, 1% BSA, 3% fetal bovine serum, 0.2% triton) for 1 hour. Then, they were incubated with the primary antibodies (Anti-Hu CD45 Alexa Fluor® 647 and EpCAM Mouse mAb Alexa Fluor® 594 Conjugate) diluted in the working buffer (PBS, 0.1% BSA, 0.3% fetal bovine serum, 0.2% triton X-100) overnight at room temperature in the dark. After several washes with the working buffer, we discarded it and the removable part of the chamber was taken off. Finally, a few droplets of Aqua-Poly/Mount (18606-100, Polysciences) were added and a cover glass was placed on top. Confocal microscopy pictures were taken (oil, 40x), and images were processed with ImageJ software.

SUPPLEMENTARY FIGURES

Supplementary Figure S1. Establishment of patient-derived organoids from ascites of GSC patients. Representative bright-field images of the different PDOs from ASC4, ASC5, ASC6 are shown. Scale bars, 100 μm.

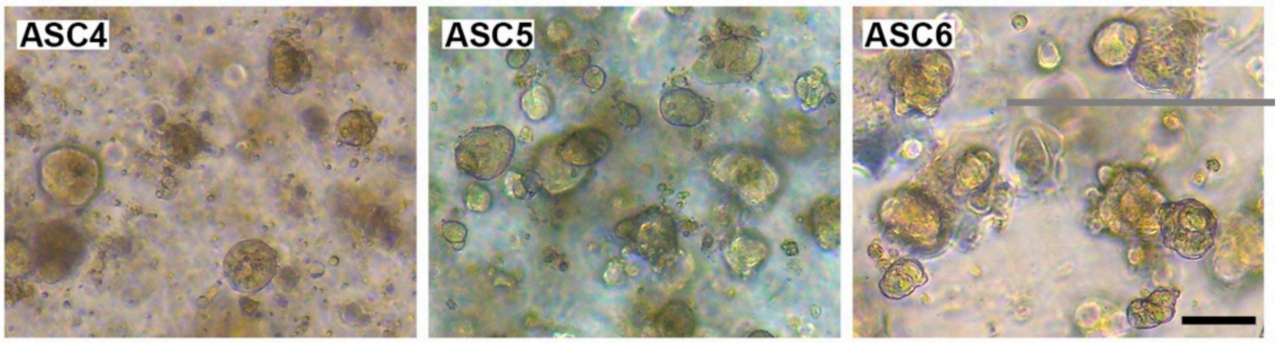

Supplementary Figure S2. Immunohistochemistry profiling of the ASC5 PDOs. The panels show representative ASC5 organoids stained with different markers of gynaecological serous carcinomas: H&E, P53, CK7, WT-1, ER, PR and PAX8, as described in the M&M section. Abundant nuclear atypia is shown in the PDOs. Scale bars, 100 µm.

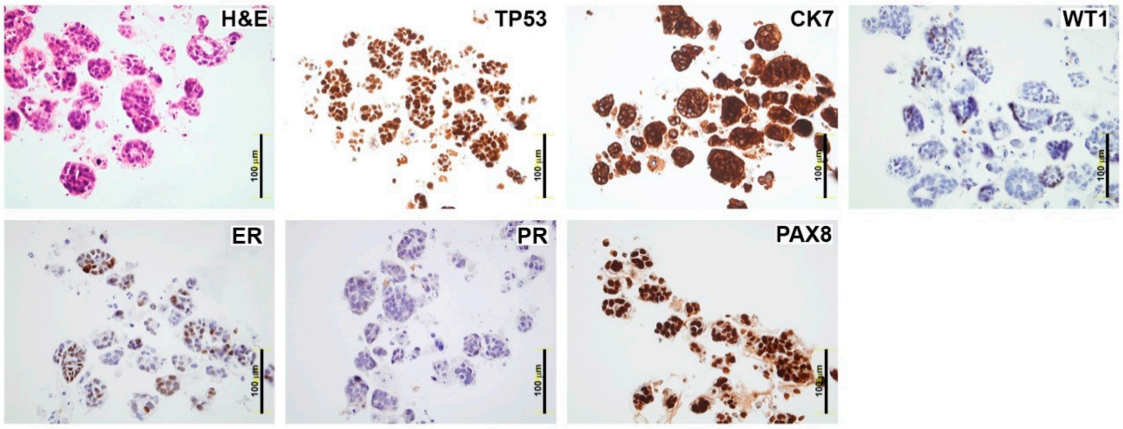

Supplementary Figure S3. Immunofluorescence characterisation of the PDOs. Representative confocal microscopy images of PDOs (ASC1, ASC5, ASC4) derived from the ascitic fluid of GSC patients, labelled with the GSC clinical markers PAX8 (nuclear) and EpCAM (membrane). Left panels show PDOs stained with PAX8, middle panels show the staining with the epithelial marker EpCAM, and the nuclear staining with DAPI; right panels are merged images. Scale bar, 100 μm.

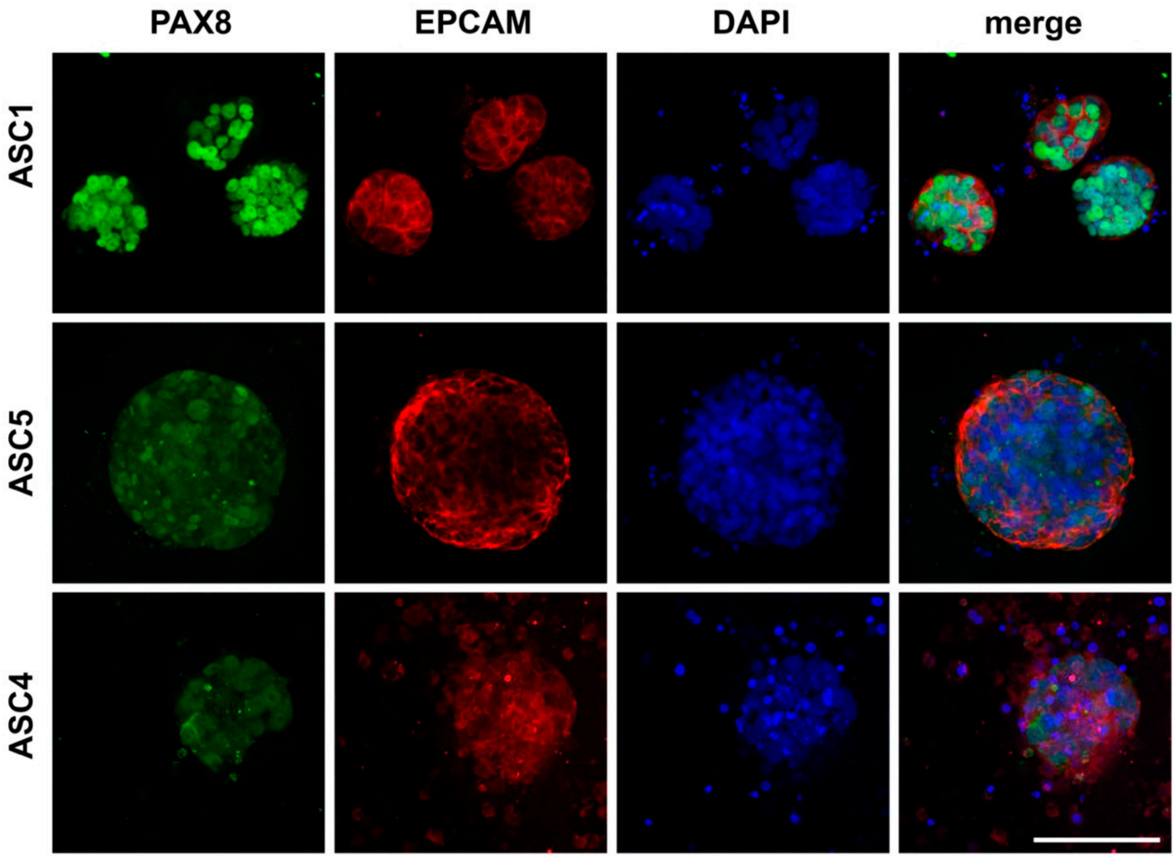

Supplementary Figure S4. Immunofluorescence characterisation of the immune-related component with CD45 in the PDOs. Representative confocal microscopy images of PDOs (ASC2, ASC1, ASC4, ASC5) derived from the ascitic fluid of GSC patients. Left panels show PDOs stained with GSC clinical marker EpCAM, middle panels show the staining with CD45 (1:200), right panels show nuclear staining with DAPI. CD45 staining showed residual presence of cells of immune and haematological origin, indicative of the purity and homogeneity of the tumoral component. Scale bar, 100 µm.

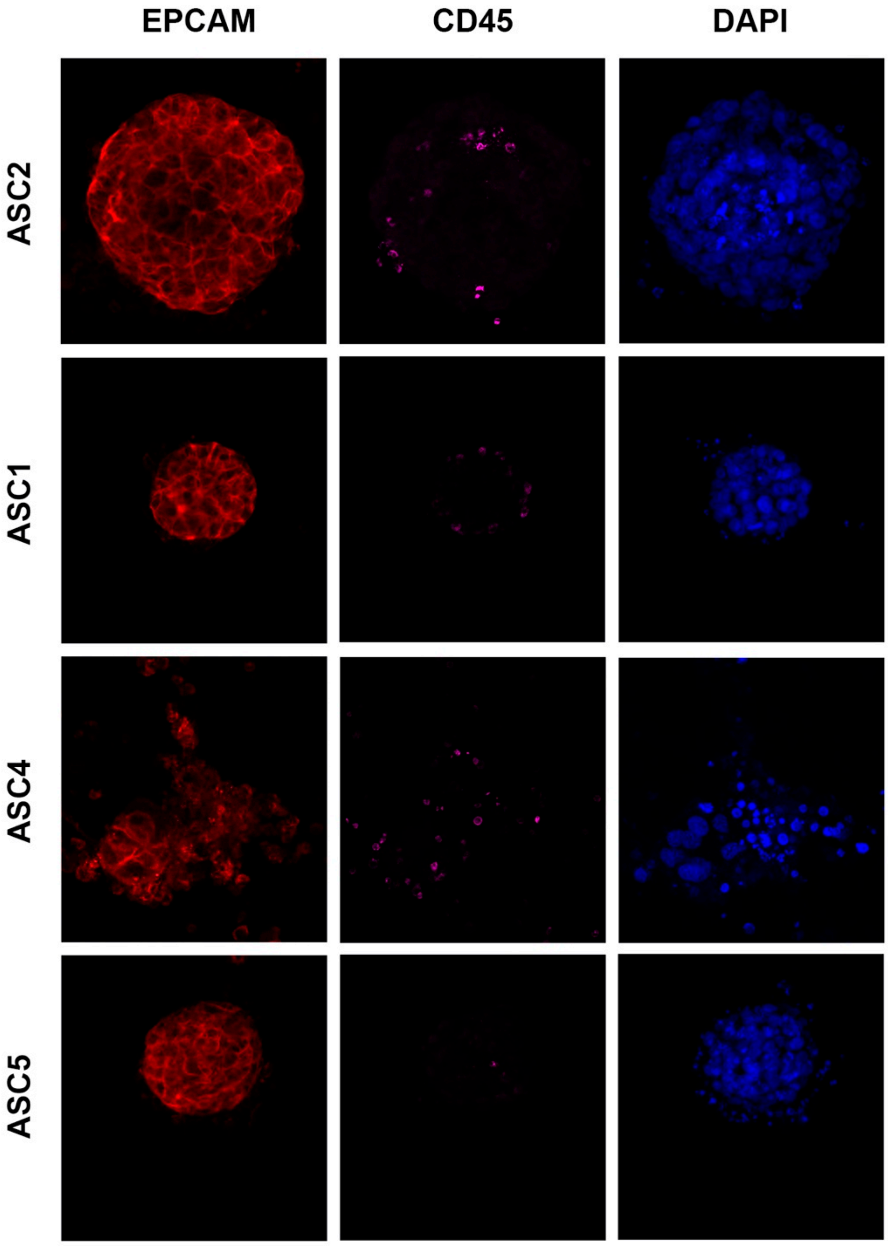

Supplementary Figure S5. Design of the drug sensitivity assays. In order to design the protocol for the drug sensitivity assays, we initially tested different concentrations of carboplatin, selected based on existing literature [10, 38]. As stated, the maximum plasma concentration of carboplatin is 135 µM, so we aimed to choose concentrations covering this range to observe the full spectrum of response, from no response to complete cell death (500µM). Representative bright-field microscopic images of treated ASC4 PDOs and the observed effects are also included.

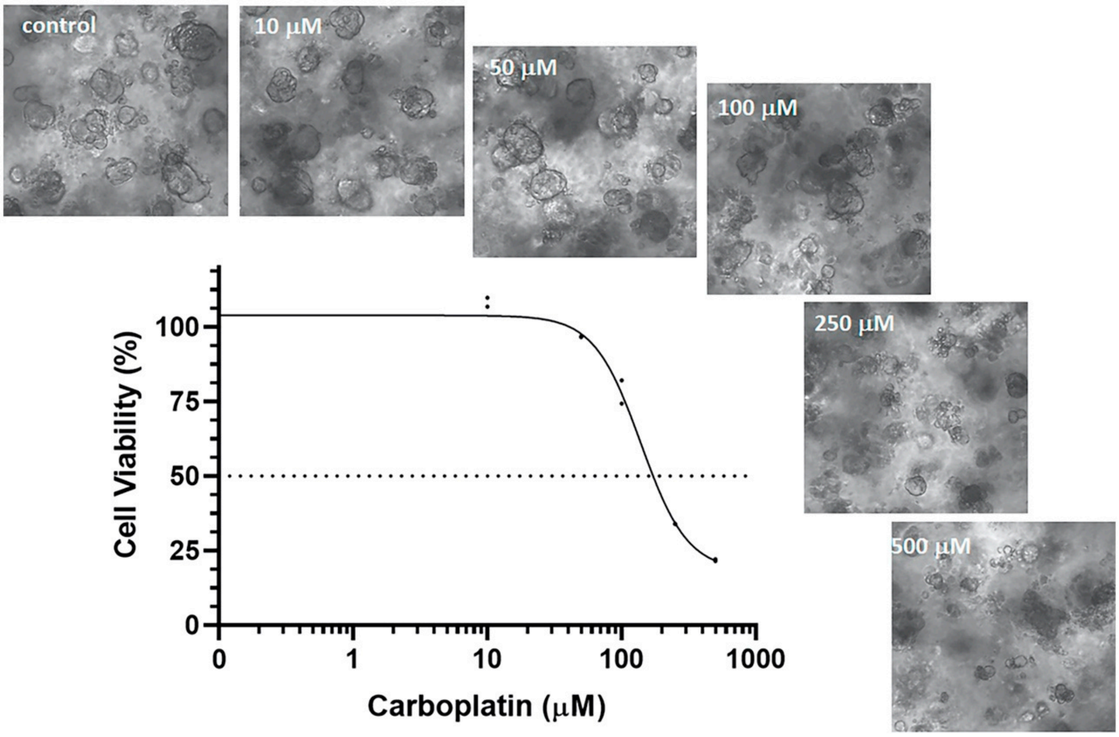

Supplementary Figure S6. Immunofluorescence analysis of Cleaved Caspase-3 in control and treated organoids. The left panels display the sensitive organoid lines ASC2 and ASC5. The right panels show activated-caspase-3 antibody staining (1:200) the resistant organoid lines ASC2 IC20-treated *in vitro* (acquired resistance *in vitro*) and ASC6 (derived from a resistant patient). In the top images, organoid controls incubated in SOM without the drug are shown, while the bottom images depict organoids treated with 10µM of carboplatin for 24 hours. As shown, while platinum-sensitive ASC2 and ASC5 PDOs presented an enhanced activated caspase-3 staining upon exposure to the platinum-based therapy, no difference was observed in the platinum-resistant ASC2 and ASC6 PDOs between treated and untreated conditions. Scale bars, 100 µm.

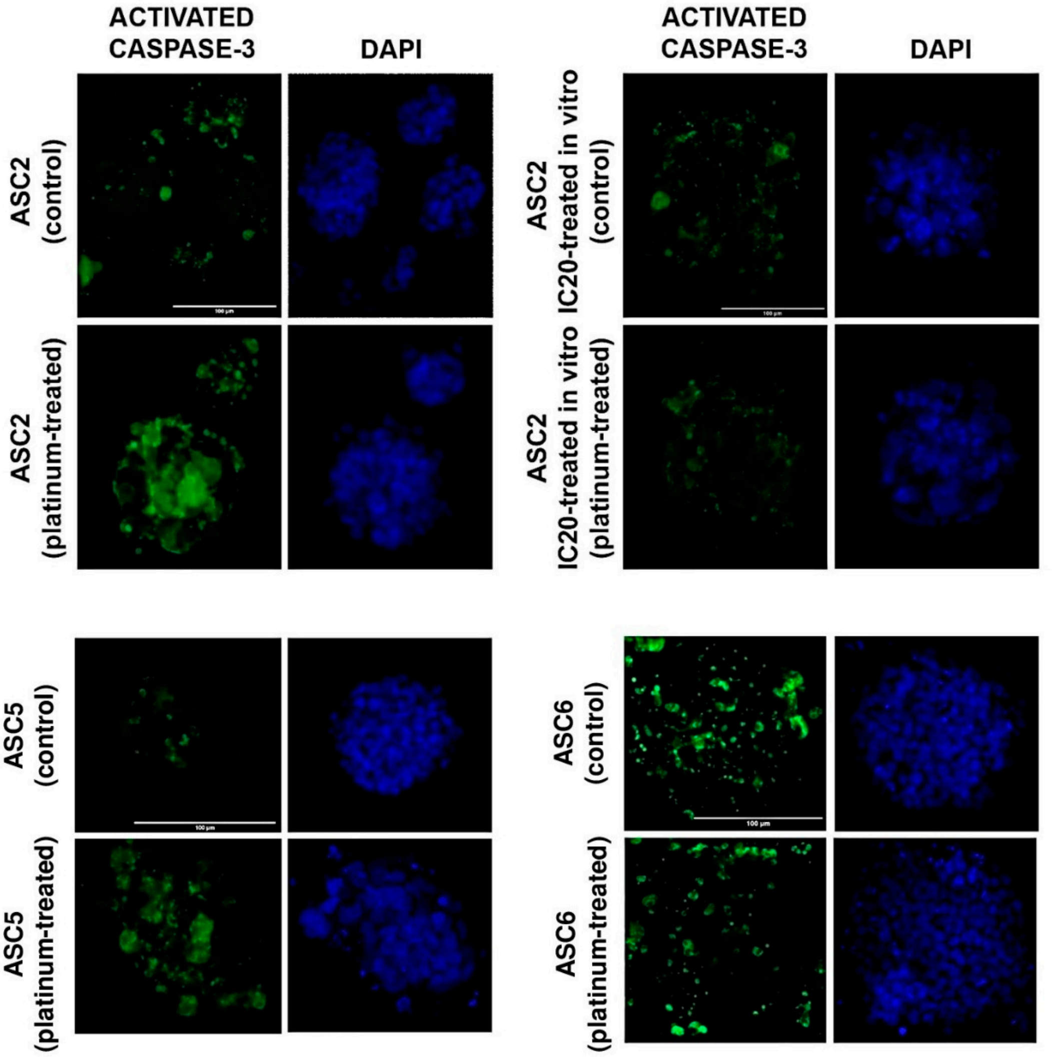

Supplementary Figure S7. Negative control for the immunofluorescence staining. The left panel show no positivity in the organoids incubated with the secondary antibody (Goat Anti-Rabbit IgG H&L Alexa Fluor® 488) but without the primary antibody, demonstrating specificity for immunofluorescence staining. The right panel show nuclear staining with DAPI. Scale bars, 100 μm.

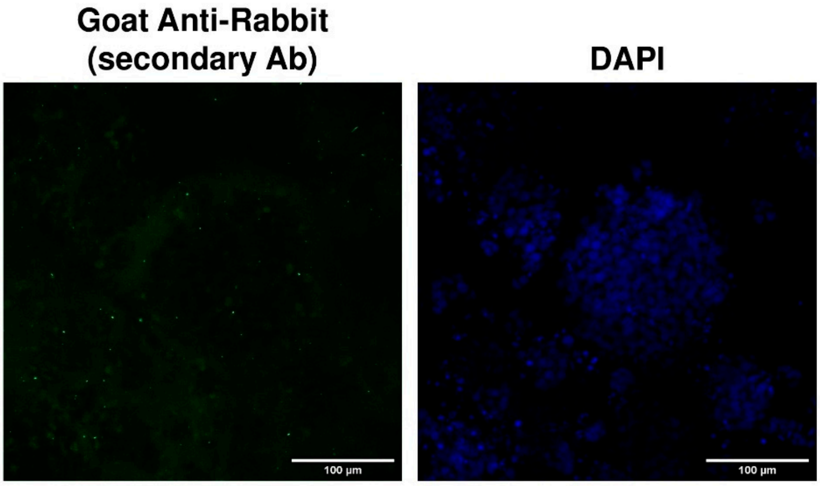

Supplement: Supplementary file 1 [file ijms-24-13208-s001.zip › ijms-2427961-supplementary.pdf]
